# Research of Optical Properties and Biocompatibility in Different Zones of Multilayered Translucent Zirconia on Hydrothermal Aging

**DOI:** 10.3390/ma17215189

**Published:** 2024-10-24

**Authors:** Ju-Hyun Kim, Ye-Jin Yang, Jin-Soo Ahn, Soo-Yeon Shin, Jung-Hwan Lee, Yu-Sung Choi

**Affiliations:** 1 Department of Prosthodontics, College of Dentistry, Dankook University, 119 Dandae-ro, Cheonan 31116, Republic of Korea; withnizi@hanmail.net; 2 Department of Biomaterials Science, College of Dentistry, Dankook University, 119 Dandae-ro, Cheonan 31116, Republic of Korea; yejin7068@naver.com; 3 Institute of Tissue Regeneration Engineering (ITREN), Dankook University, 119 Dandae-ro, Cheonan 31116, Republic of Korea; 4 Dental Research Insitute and Biomaterials Science, Dentistry, Seoul National University, Seoul 03080, Republic of Korea; ahnjin@snu.ac.kr; 5 Department of Nanobiomedical Science & BK21 PLUS NBM Global Research Center for Regenerative Medicine Research Center, Dankook University, 119 Dandae-ro, Cheonan 31116, Republic of Korea; 6 Mechanobiology Dental Medicine Research Center, Dankook University, Cheonan 31116, Republic of Korea; 7 UCL Eastman-Korea Dental Medicine Innovation Centre, Dankook University, 119 Dandae-ro, Cheonan 31116, Republic of Korea; 8 Cell & Matter Institute, Dankook University, Cheonan 31116, Republic of Korea; 9 Department of Regenerative Dental Medicine, School of Dentistry, Dankook University, Cheonan 31116, Republic of Korea

**Keywords:** zirconia, hydrothermal aging, optical property, biocompatibility, surface property, yttrium oxide

## Abstract

Objective: We assessed the changes in optical properties and biocompatibility of transition zones in multilayered translucent monolithic zirconia exposed to prolonged hydrothermal aging and compared the results to those with different yttrium oxide contents. Materials and Methods: Four types of zirconia blocks from IPS e.max ZirCAD were used: 3Y-TZP e.max ZirCAD LT (ZL), 4Y-TZP e.max ZirCAD MT (ZM), 5Y-TZP e.max ZirCAD MT Multi (ZT), and 3Y/5Y-TZP e.max ZirCAD Prime (ZP). A total of 120 specimens (15.0 mm diameter and 1.5 mm height) were fabricated and divided into three groups (*n* = 10). The aging process for the specimens was conducted in an autoclave set to 134 °C and 0.2 MPa, with durations of 0 h (control), 5 h (first aged), and 10 h (second aged). The optical properties and biocompatibility were analyzed, followed by a statistical analysis of the data (α = 0.05). Results: Before and after aging, ZL and ZP exhibited the lowest color changes. ZT exhibited the highest average transmittance and translucency parameter values, while ZL had the lowest. The water contact angle test showed the highest value in ZM and lowest in ZL across all the aging stages. ZL, ZM, and ZP showed a considerable decrease in the water contact angle; however, ZT did not. A cell counting kit-8 assay showed ZL had the highest value, while ZM had the lowest. A filamentous actin test exhibited the highest value in ZL and lowest in ZM. In the vinculin analysis, ZL and ZT exhibited the lowest values, whereas ZM and ZP had the highest. Conclusion: 3Y/5Y-TZP exhibited a balanced performance across critical parameters, such as color stability, translucency, and biocompatibility, aligning with 3Y-TZP. While 5Y-TZP demonstrated superior translucency, it confirmed the lowest color stability, whereas 3Y-TZP achieved the highest biocompatibility. These properties provide clinicians with a reliable material option that ensures superior esthetic outcomes and long-term prognosis, ultimately contributing to improved patient satisfaction and clinical longevity.

## 1. Introduction

In the field of dental prosthetics, there exists a continuous demand for materials that offer esthetic excellence and biocompatibility with human tissues. Advancements in computer-aided design and computer-aided manufacturing (CAD/CAM) systems [1,2,3] have enabled the creation of precise and esthetically appealing dental restorative materials [4,5,6]. Among the available materials (resin, ceramic, and zirconia), zirconia has garnered considerable attention as a restorative material in dental material studies owing to its superior mechanical properties, high fracture resistance, and excellent biocompatibility and esthetics [7,8]. Despite its strength, 3Y-TZP (3 mol% yttria-stabilized tetragonal zirconia polycrystal) is opaque and does not perfectly replicate natural teeth [9,10,11]. To address this, translucent zirconia with an increased yttria content was introduced, and multilayered translucent zirconia with varying yttria contents was also developed [12].

Multilayered translucent zirconia differs from conventional monolithic zirconia in that it features layers that simulate the enamel, dentin, and gradation layers, thereby enabling it to replicate the color and translucency of natural teeth from the cervical to occlusal surfaces [13]. The development of multilayered translucent zirconia, which address high opacity while achieving esthetic goals, has resulted in an increase in its usage [14,15]. Studies have shown that multilayered translucent zirconia provides superior color reproduction compared to conventional translucent zirconia [16,17].

Yttria (Y_2_O_3_) is an additive utilized to affect the tetragonal phase and optical properties of zirconia, respectively [18]. Its content in zirconia affects the translucency and mechanical strength of the material, with increased levels enhancing its translucency owing to a higher proportion of the cubic phase at room temperature [16,19]. This increase hampers the birefringence that occurs at the grain boundaries, thereby increasing the translucency. However, it also reduces the tetragonal phase, which contributes to the mechanical strength. As the sintering temperature increases, the crystal size increases, thereby degrading the mechanical properties (fracture resistance and flexural strength) better than conventional monolithic zirconia and resulting in color stability variations [9].

According to more recent citations, advancements have been made by incorporating stabilizers, such as alumina and cerium oxide, which have shown improved resistance to low-temperature degradation (LTD). These materials demonstrate enhanced toughness and better long-term stability under hydrothermal conditions [20]. Recent advancements in dental materials include hybrid compounds containing zirconium and phosphorus, synthesized via methods like the sol–gel process. These materials show enhanced biocompatibility, with applications in dentistry, as demonstrated by Khaled et al. [21]. Such hybrid materials offer improved biocompatibility and stability for dental restorations. These advancements, by evaluating the biocompatibility and optical properties of zirconia, support its use as an innovative material for dental applications.

Ensuring stability against low-temperature degradation (LTD) is crucial when employing zirconia. This phenomenon occurs as zirconia transitions from the tetragonal to the monoclinic phase at low temperatures. Because monoclinic crystals are larger than tetragonal crystals, this phase change causes volumetric expansion, thereby forming microcracks starting from the surface of the zirconia and reducing its strength [22]. Aging can accelerate deterioration via increased surface roughness, enhanced wear, decreased fracture toughness, and reduced hardness [23,24].

Hydrothermal aging is crucial for evaluating the long-term stability and reliability of zirconia, particularly considering the exposure to the oral environment with temperature fluctuations and high humidity [25]. Zirconia is susceptible to phase transformation from a tetragonal to a monoclinic structure. Hydrothermal aging allows for the evaluation of the microstructural stability of a material relative to crack growth and decreased strength during this transition [26]. Moisture may accelerate crack propagation and grain growth within the zirconia, thereby adversely affecting the mechanical properties [27].

From a biocompatibility perspective, dental restorative materials are in direct contact with oral soft tissues, particularly the gingiva. Human gingival fibroblasts (HGFs) are the principal cell types in gingival tissues and are crucial in gingival health and regeneration [28]. Evaluating their interaction with zirconia is key in assessing the biocompatibility of this material and predicting its long-term effects on periodontal tissues [29]. In prosthodontics, research on the interaction between gingival fibroblasts and dental materials is crucial, as these cells play a key role in the soft tissue’s biological response to biomaterials in the oral environment. The behavior of fibroblasts significantly influences the integration and long-term success of prosthetic restorations [30]. Optical properties are vital for the esthetic appeal and long-term success of dental prostheses [31]. Prosthetic materials must demonstrate considerable resistance to discoloration, even after prolonged exposure to various staining agents, such as coffee, tea, and wine. Further, the color stability of dental zirconia is influenced by its interaction with light and the oral environment. Understanding the effect of the yttria content on the optical properties and biocompatibility of multilayered translucent zirconia is key for optimizing both esthetics and stability in dental prostheses.

To provide a broader understanding of the findings, it is crucial to consider how the processing of other dental bioceramics affects their color stability. Materials such as lithium disilicate and alumina ceramics are also commonly used in dental applications and undergo similar processing and environmental exposures as zirconia. Lithium disilicate, which is known for its superior translucency and esthetic qualities, can undergo significant color changes due to surface treatments and prolonged exposure to humid conditions. Similarly, alumina ceramics, though characterized by high mechanical strength, are susceptible to variations in color stability after prolonged thermal and mechanical processing. These findings underline the importance of considering the impact of processing techniques on long-term color stability, not only for zirconia but also for other widely used dental ceramics [32].

Previous research on yttria-stabilized zirconia has focused on its mechanical properties, with less attention on the impact of varying yttria content on the optical properties and biocompatibility of multilayered translucent zirconia [33]. This study addresses this gap by investigating the effect of different yttria concentrations on the optical properties and biocompatibility, particularly considering the effects of aging. It focuses on the transition zone (3Y/5Y-TZP) of multilayered translucent zirconia, which was developed using gradient technology, subjected to long-term hydrothermal aging. This study also compares the stability of these properties in the transition zone to other zones of conventional and multilayered translucent zirconia, including 3Y-TZP, 4Y-TZP, and 5Y-TZP.

We aim to evaluate the changes in optical properties and biocompatibility of transition zones in multilayered translucent monolithic zirconia by comparing the results to those with different yttrium oxide content on hydrothermal aging.

## 2. Materials and Methods

### 2.1. Specimen Preparation

Four types of zirconia from IPS e.max ZirCAD (IPS e.max ZirCAD) were utilized as follows (Table 1): IPS e.max ZirCAD LT, IPS e.max ZirCAD MT, IPS e.max ZirCAD MT Multi, and IPS e.max ZirCAD Prime. e.max ZirCAD LT and IPS e.max ZirCAD MT are conventional monolithic zirconias, which contain 3Y-TZP (3 mol% yttria-stabilized tetragonal zirconia polycrystal). IPS e.max ZirCAD MT Multi and IPS e.max ZirCAD Prime variants are multilayer blocks designed with incisal, transition, and dentin zones to replicate the natural gradation and translucency of dental enamel and dentin [34]. In IPS e.max ZirCAD MT Multi, the incisal region utilizes translucent 5Y-TZP zirconium oxide for enhanced translucency, while the dentin area uses opaquer 4Y-TZP zirconia oxide to replicate the natural opacity of dentin. IPS e.max ZirCAD Prime is fabricated by blending 3Y-TZP and 5Y-TZP, thereby offering unique properties for dental restorations. Specimens of IPS e.max ZirCAD MT Multi were from the 5Y-TZP incisal zone, while those of IPS e.max ZirCAD Prime were from the 3Y/5Y-TZP transition zone, with zirconia specimens produced for each region based on the yttria content.

The samples underwent sintering at 1500 °C, following the manufacturer’s guidelines for zirconia-based ceramics (Ivoclar, Schaan, Liechtenstein, 2024), while considering a 20% shrinkage rate. A total of 120 specimens were fabricated, with each material forming circular specimens with dimensions of 15.0 and 1.5 mm in diameter and thickness, respectively. The specimens were prepared with a precision milling machine and polished on both sides using a grinding process that utilized a 6 μm diamond slurry and a 1 μm grit size. This procedure ensured a uniform thickness of 1.5 ± 0.05 mm after polishing. After the finishing and polishing steps, the dimensions of all the specimens were evaluated using digital calipers. To induce reverse phase transformation, heat treatment was conducted for 1 h in the temperature range of 900–1000 °C using a Programat EP5000 furnace (Ivoclar, Schaan, Liechtenstein). After the heat treatment, all the specimens were rinsed in distilled water using an ultrasonic cleaner for 10 min, then air-dried at room temperature (21–23 °C) for 24 h. Subsequently, all the dried specimens were categorized into three groups (*n* = 10 per group) according to the following criteria: the control group with no aging, the first aging group, and the second aging group (Table 2).

### 2.2. Hydrothermal Aging Process

The experimental protocol for aging the zirconia specimens was designed to simulate the long-term conditions encountered in clinical scenarios. The aging process is crucial for assessing the durability of materials over extended periods. Hydrothermal aging was conducted in an autoclave, which is a key component in the aforementioned procedure. The aging protocol was divided into two phases. In the first phase, the specimens were exposed to 134 °C and 0.2 MPa for 5 h. The second phase extended this exposure to 10 h under similar conditions, thereby simulating a more prolonged clinical lifespan and further accelerating the aging process. Conversely, the control specimens were maintained without any aging treatment. This allowed for a comparison to assess the effects of the aging process on the material properties. One hour of exposure at 134 °C and 0.2 MPa is equivalent to approximately 3–4 years in a clinical oral environment [35,36].

### 2.3. Comparative Analysis of Optical Properties

To assess the optical characteristics of the various zirconia specimens, this study conducted a detailed comparative analysis using advanced colorimetric and translucency assessments. The principal tool used was a spectrophotometer, which provided precise and consistent color and translucency measurements under controlled conditions.

#### 2.3.1. Color Analysis

The colors of all the specimens were analyzed using CIELab color space parameters. The L* (lightness), a* (red–green axis), b* (blue–yellow axis), C* (chroma), and h (hue) values were measured for each specimen. These parameters quantify the color lightness spectrum, red–green axis, and blue–yellow scale, thereby providing a detailed color profile for each sample [37]. This study examined the variances in these parameters before and after hydrothermal aging to simulate the long-term effects of clinical conditions on the esthetic properties of the material.

#### 2.3.2. Translucency and Contrast Ratio

Translucency is a key factor in the esthetic integration of dental materials with natural teeth. This was quantified by calculating the average transmittance (At) for each specimen [38]. Additionally, the contrast ratio (CR) was determined using Y_b_ and Y_w_ measured against black and white backgrounds, with the CR values ranging from 0 (total translucency) to 1 (complete opacity). This measurement is crucial for assessing how zirconia blends with its surrounding under different lighting conditions, as expressed as follows [16]:

(1) $$CR=\frac{Y_{b}}{Y_{w}}$$

#### 2.3.3. Perceptible Color Difference

To enhance the comparative analysis, this study calculates the perceptible color differences between the specimens using the ΔE and ΔE_00_ formulas [39]. These metrics are crucial for determining the clinical acceptability of materials based on visible color changes after aging. The ΔE values provide a basic Euclidean distance between color measurements, whereas ΔE_00_ offers a more precise analysis by accounting for perceptual non-uniformities in the color space, thereby enhancing the clinical relevance of the findings.

(2) $$\Delta E=\sqrt{{\left(L_{1}-L_{2}\right)}^{2}+{\left(a_{1}-a_{2}\right)}^{2}+{\left(b_{1}-b_{2}\right)}^{2}}$$

(3) $${∆E}_{00}={\left[{\left(\frac{\Delta L^{\prime }}{K_{L}S_{L}}\right)}^{2}+{\left(\frac{\Delta C^{\prime }}{K_{c}S_{C}}\right)}^{2}+{\left(\frac{\Delta H^{\prime }}{K_{H}S_{H}}\right)}^{2}+R_{T}\left(\frac{\Delta C^{\prime }}{K_{C}S_{C}}\right)+(\frac{\Delta H^{\prime }}{K_{H}S_{H}})\right]}^{1/2}$$

### 2.4. Comparative Analysis of Surface Properties

This section of the study examines the comparative analysis of the biocompatibility of different dental materials, notably on zirconia-based specimens, using various cellular and molecular assays to evaluate their interactions with human gingival fibroblasts (HGFs) [40].

#### 2.4.1. Surface Characterization

Biocompatibility begins with analyzing the surface characteristics, which is crucial for understanding how materials interact with biological tissues [41]. Atomic force microscopy (AFM) provided detailed insights into the surface topography and roughness of the specimens, which are crucial for cellular responses. Measurements of the roughness parameters, including R_a_, R_q_, S_a_, and S_q_, provided insights into the microscale texture that impacts cell adhesion and proliferation.

#### 2.4.2. Hydrophilicity Assessment

The wettability of the materials, assessed via water contact angle measurements, indicates the ability of the surface to support cell adhesion, which is a key component of biocompatibility [42]. The sessile drop method was applied to evaluate the static contact angle, thereby providing an indirect assessment of the biochemical properties of the surface and its potential interaction with biological fluids.

### 2.5. Comparative Analysis of Cellular Properties

#### 2.5.1. Cell Culture

The HGFs were isolated from gingival fragments acquired during surgical tooth extractions from patients in their twenties of the Department of Oral and Maxillofacial Surgery, Dental Hospital, Dankook University, Republic of Korea [43,44].

This study was carried out in compliance with the ethical standards outlined by the Dankook University Dental Hospital Institutional Review Board (DKUDH IRB). The study protocol was approved by the DKUDH IRB on 24th July 2023 (IRB No.: DKUDH IRB 2023-06-001). All the procedures were conducted according to the relevant laws and institutional guidelines, and informed consent was acquired from all the participants involved in this study. Fresh gingival fragments were stored in 5 mL of Hank’s balanced salt solution containing 1% penicillin/streptomycin. To minimize contamination risks, the fragments were immersed for 1–2 min in phosphate-buffered saline containing 2% PS and later cleaned using 1X PBS to eliminate excess PS. Blood vessels were removed with scissors and forceps, and the fragments were later immersed in a solution containing 2 mg/mL collagenase type I and 4 mg/mL dispase II for 1 h at 37 °C in a water bath for dissociation. The gingival epithelial layers were isolated, diced into approximately 1 × 1 mm^2^ fragments, and transferred to culture dishes. The tissues were cultured in less than 2 mL of minimum essential medium alpha modification (αMEM; LM008-01, Welgene, Gyeongsan, Korea) supplemented using 10% fetal bovine serum 100 mM non-essential amino acids solution (100X; Gibco™, Waltham, MA, USA), 2 mM L-glutamine (GlutaMAX™-1 100X; Gibco™, Waltham, MA, USA), 55 μM 2-mercaptoethanol (1000X; Gibco™, Waltham, MA, USA), and 1% PS. The culture medium was replaced after 48 h of initial incubation and every 2–3 days thereafter for 10–14 days. The primary cell colonies were collected and sub-cultured. Four groups of dental IPS e.max ZirCAD blocks were placed on untreated plates and seeded using the HGFs. The experiments were performed 24 h after seeding, using the HGFs at passages 5–10.

#### 2.5.2. Viability Assay

The viability and proliferation of the HGFs were assessed with cell counting kit-8, which analyzes mitochondrial dehydrogenase activity in living cells [45]. Four groups of samples were placed in untreated 24-well plates and seeded using the HGFs (5 × 10^4^ cells/mL). After 24 h of incubation, each well was cleaned using phosphate-buffered saline (PBS) to remove unattached cells and debris. Further, 1 mL of 10% CCK-8 working solution was added, followed by incubation at 37 °C for 2 h. After incubation, only 100 μL of the medium was transferred to each well in the 96-well plate. The absorbance at 450 nm was analyzed with a Varioskan LUX multimode microplate reader (Thermo Fisher Scientific, Waltham, MA, USA) to quantify the cell viability based on the mitochondrial dehydrogenase activity.

#### 2.5.3. Live/Dead Assay

Four groups of samples were placed in untreated 24-well plates and seeded with the HGFs at a density of 5 × 10^4^ cells/well, with three replicates for statistical validity. After 24 h of incubation for cellular adhesion and proliferation, the samples were washed using PBS. The live/dead solution, comprising 2 μM calcein-AM (C1430, Invitrogen, Waltham, MA, USA) and 4 µM ethidium homodimer-1 (EthD-1, E1169, Invitrogen, Waltham, MA, USA), was prepared according to the instructions of the manufacturer and added to each well. The samples were incubated at 37 °C for 30 min in a 5% CO_2_ atmosphere for optimal staining. After incubation, cells images were captured with a fluorescence microscope. Dead cells were stained red using EthD-1, while live cells were stained green using calcein-AM [46].

#### 2.5.4. Analysis of Cellular Cytoskeletal Organization and Morphology

To analyze the cytoskeletal organization and morphological characteristics of the HGFs, the cells were seeded at a density of 2 × 10^4^ per well into untreated 24-well plates cells. After 24 h of incubation, the HGFs were cleaned thrice using PBS and fixed using 4% paraformaldehyde (PFA; T & I Biotechnology, Seoul, Republic of Korea) for 15 min at room temperature. Filamentous actin (F-actin) was later stained using Alexa Fluor 546-conjugated phalloidin (#A22283, Invitrogen, Waltham, MA, USA) for 30 min at room temperature, while the nuclei were stained with SYTOX™ Green Nucleic Acid Stain (S7020, Invitrogen, Waltham, MA, USA) for 10 min at room temperature [47]. The stained cells were imaged using a fluorescence microscope (IX71, Olympus, Tokyo, Japan) to capture high-resolution images. These images were analyzed with ImageJ software (Version 1.54; National Institutes of Health, Bethesda, MD, USA) to measure the F-actin intensity, area, circularity, length, and width.

#### 2.5.5. Analysis of Focal Adhesion

To investigate the focal adhesion properties of the HGFs, immunofluorescence labeling focusing on vinculin was conducted. The cells were seeded into untreated 24-well plates at a density of 2 × 10^4^ cells/well. After 24 h of incubation, the HGFs were washed using HBSS containing Ca^2+^ and Mg^2+^ (LB 003-02, Welgene, Gyeongsan, Republic of Korea) and fixed in 4% PFA (Tech & Innovation, Gangwon, Republic of Korea) for 15 min at room temperature. After fixation, the cells were permeabilized with a 0.2% Triton X-100 solution for 10 min and later blocked with 1% bovine serum albumin fraction for 1 h. Further, the cells were incubated overnight at 4 °C with rabbit anti-vinculin first antibody [48]. After washing them thrice using HBSS, they were stained with a fluorescein isothiocyanate-conjugated secondary antibody for 4 h at room temperature. The samples were examined under a fluorescence microscope (IX71). A quantitative assessment of the vinculin fluorescence area and intensity was conducted using ImageJ software.

### 2.6. Statistical Analyses

Statistical analyses were conducted using IBM SPSS Statistics (version 23.0 and 29.0.1.0). The normality was evaluated with the Shapiro–Wilk test, while the homogeneity of variance was assessed using Levene’s test. The key surface properties, such as surface roughness and contact angle, coupled with biological parameters (F-actin intensity, cell area, and vinculin intensity) were analyzed before and after hydrothermal aging. This analysis involved one-way ANOVA, independent samples t-tests, and Tukey HSD post hoc tests for multiple comparisons between groups. Variables such as CIELab values, translucency parameter, and color stability were also examined before and after hydrothermal aging using similar statistical methods to further understand the interaction between the material properties and aging processes. A significance level of *p* < 0.05 was maintained to identify statistically considerable differences (α = 0.05).

## 3. Results

### 3.1. CIE Lab* Color Space

This study investigated the CIE Lab* color space parameters (L*, a*, and b*), chroma (C*), and hue (h*) values to evaluate the color stability and optical characteristics of different types of zirconia based on the yttrium oxide content during hydrothermal aging (Table 3).

### 3.2. Color Stability

**Figure 1:**
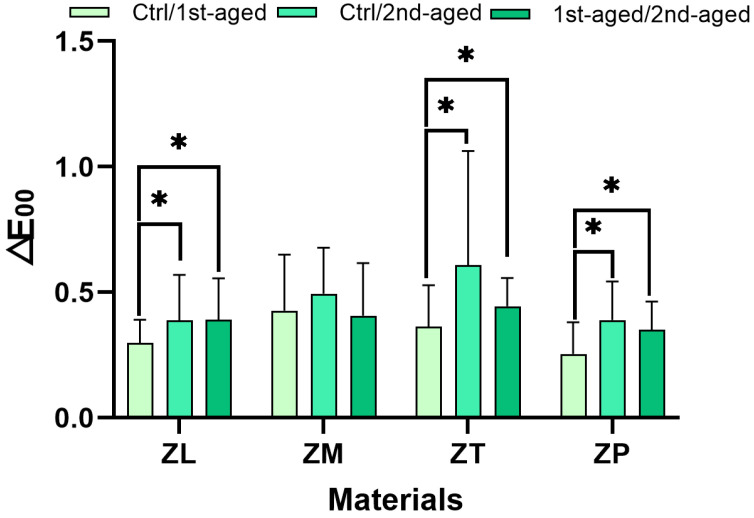
The mean values of the color variations as measured by ΔE_00_ (black background, SCI) of all the specimens in the groups. ZL, monolayered zirconia with 3 mol% yttria (3Y-TZP), IPS e.max ZirCAD LT; ZM, monolayered zirconia with 4 mol% yttria (4Y-TZP), IPS e.max ZirCAD MT; ZT, incisal zone of multilayered zirconia with 5 mol% yttria (5Y-TZP), IPS e.max ZirCAD MT Multi; ZP, transition zone of multilayered zirconia with 3 and 5 mol% yttria (3Y/5Y-TZP), IPS e.max ZirCAD Prime. All the data are presented as mean ± standard deviation values. * denotes a considerable difference at *p* < 0.05.

The mean values of the color variations as measured by ΔE_00_ for all the experimental groups are shown in Figure 1 and Table 4. Here, the ΔE_00_ metric is adjusted to correspond with human visual perception. After the first aging, ZM showed the highest color changes (ΔE_00_ = 0.43 ± 0.22), while ZT exhibited the highest color changes after the second aging (ΔE_00_ = 0.61 ± 0.45). ZP exhibited the lowest color changes after first aging (ΔE_00_ = 0.25 ± 0.13) and second aging (ΔE_00_ = 0.39 ± 0.15), thereby indicating ZL and ZP materials are less prone to color changes. Overall, the data indicate that yttrium oxide content considerably impacts the color stability of dental materials during hydrothermal aging. The ZM materials exhibited the most considerable changes between the control and first aging (for ΔE_00_), while the ZT materials exhibited the most considerable variations between the control and second aging (for ΔE_00_), and ZP exhibited the lowest ΔE_00_ values.

### 3.3. Transmittance

**Figure 2:**
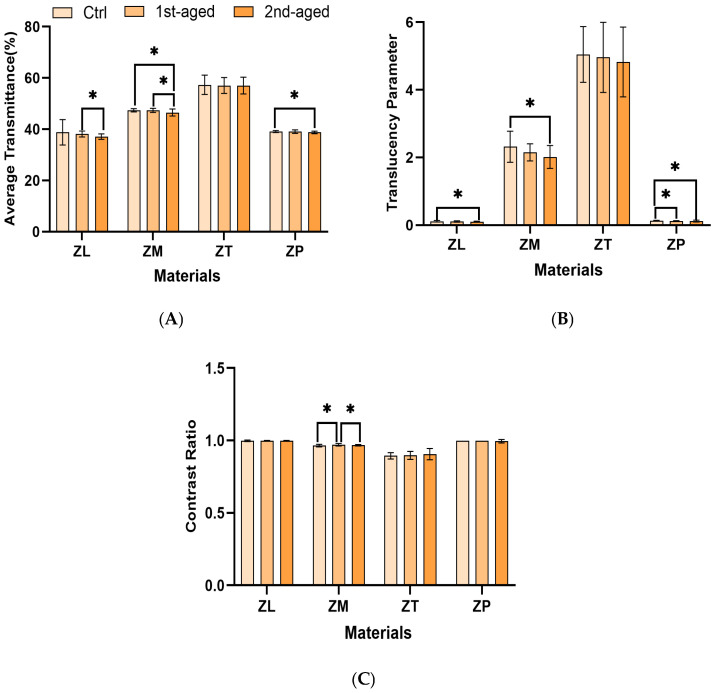
The mean values of the optical properties of all the specimens in the groups. (**A**) Average transmission; (**B**) translucency parameter; and (**C**) contrast ratio. ZL, monolayered zirconia with 3 mol% yttria (3Y-TZP), IPS e.max ZirCAD LT; ZM, monolayered zirconia with 4 mol% yttria (4Y-TZP), IPS e.max ZirCAD MT; ZT, incisal zone of multilayered zirconia with 5 mol% yttria (5Y-TZP), IPS e.max ZirCAD MT Multi; ZP, transition zone of multilayered zirconia with 3 and 5 mol% yttria (3Y/5Y-TZP), IPS e.max ZirCAD Prime. All the data are presented as mean ± standard deviation values. * denotes a considerable difference at *p* < 0.05.

This study on the transmittance in zirconia dental materials provided a detailed analysis of the optical properties, including the At (average transmission), TP (translucency parameter), and CR (contrast ratio) across different groups of zirconia specimens (Figure 2 and Table 5). ZT exhibited the highest At and TP values, indicating its superior translucency. In contrast, ZL showed the lowest At and TP values, suggesting lower translucency, which could adversely affect the esthetic outcome in dental applications. For CR, ZL had the highest value, indicating greater opacity, and ZT had the lowest, indicating a closer resemblance to a natural tooth. ZP showed similar patterns to ZL.

### 3.4. Surface Roughness

**Figure 3:**
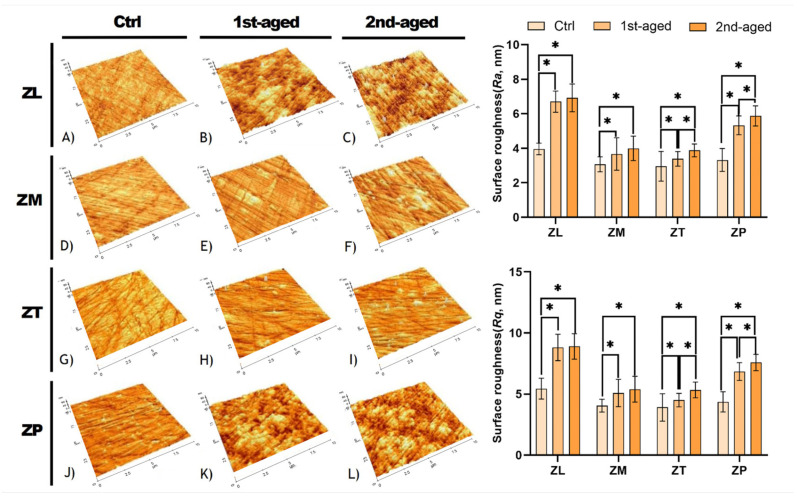
AFM images of mean ± standard deviation values and statistical analysis of the surface roughness (*R_a_* and *R_q_*) of all the specimens in the groups. (**A**) ZLC, control group of ZL; (**B**) ZLAF, first-aged group of ZL; (**C**) ZLAS, second-aged group of ZL; (**D**) ZMC, control group of ZM; (**E**) ZMAF, first-aged group of ZM; (**F**) ZMAS, second-aged group of ZM; (**G**) ZTC, control group of ZT; (**H**) ZTAF, first-aged group of ZT; (**I**) ZTAS, second-aged group of ZT; (**J**) ZPC, control group of ZP; (**K**) ZPAF, first-aged group of ZP; and (**L**) ZPAS, second-aged group of ZP. ZL, monolayered zirconia with 3 mol% yttria (3Y-TZP), IPS e.max ZirCAD LT; ZM, monolayered zirconia with 4 mol% yttria (4Y-TZP), IPS e.max ZirCAD MT; ZT, incisal zone of multilayered zirconia with 5 mol% yttria (5Y-TZP), IPS e.max ZirCAD MT Multi; ZP, transition zone of multilayered zirconia with 3 and 5 mol% yttria (3Y/5Y-TZP), IPS e.max ZirCAD Prime. All the data are presented as mean ± standard deviation values. * denotes a considerable difference at *p* < 0.05.

Representative AFM images of all the groups are presented in Figure 3. The mean values, standard deviations, and statistical analysis results for the surface roughness (*R_a_*, *R_q_*, *S_a_*, and *S_q_*) are presented in Table 6. An analysis of the surface roughness parameters using atomic force microscopy (AFM) showed considerable differences among the control, first-aged, and second-aged samples. ZL exhibited the highest *R_a_* (3.95 ± 0.34) nm, *R_q_* (5.44 ± 0.85) nm, *S_a_* (4.41 ± 1.25) nm, and *S_q_* (5.86 ± 1.62) nm in the control, while ZT exhibited the lowest values for all these parameters across all the ages. With aging, all the groups experienced an increase in surface roughness. Statistically significant differences were exhibited when comparing the control group with the first-aged group for all the parameters (*R_a_*, *R_q_*, *S_a_*, and *S_q_*) across ZL, ZM, ZT, and ZP (*p* < *0*.05). Additionally, significant differences were noted between the first and second ages for ZT and ZP. The independent t-test results confirmed considerable changes in roughness between the control and both age groups as the aging time increased (*p* < 0.05).

### 3.5. Water Contact Angle

This test revealed the highest and lowest values for ZM and ZL, respectively, across all the aging stages. ZL, ZM, and ZP exhibited a significant decrease in the contact angle than in the control group (*p* < 0.05). Further decreases were observed when comparing the control group to the second-age group (*p* < 0.05), thereby indicating a progressive increase in hydrophilicity over time. This implies that aging treatments enhance the hydrophilic properties of the material. Notably, ZT did not exhibit any considerable changes in the contact angle throughout the aging process. All the groups except ZT exhibited a considerable decrease in the contact angles in the first-aged samples than the control. ZL was the only group that exhibited a statistically considerable change in hydrophilicity between the first and second aging periods. Regarding the remaining groups, the impact of the first aging process on hydrophilicity was not considerably altered by the second aging process. ZP exhibited contact angle values similar to those of ZT (Table 7).

### 3.6. CCK-8 Assay

**Figure 4:**
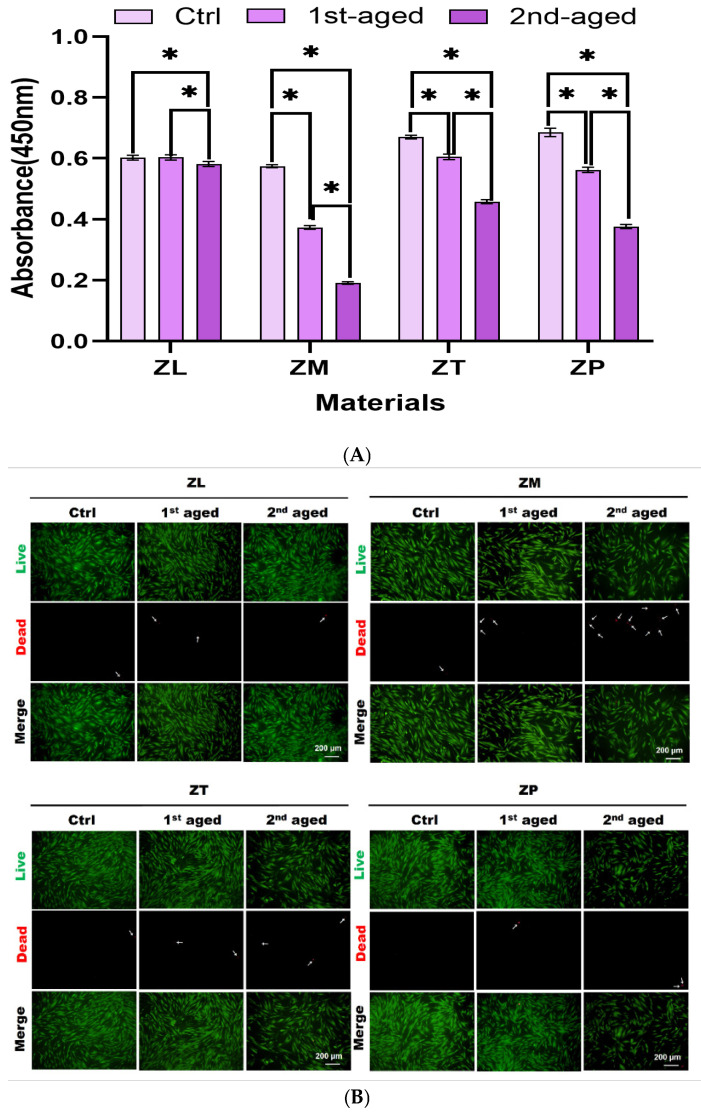
HGF viability after IPS e.max ZirCAD aging. (**A**) The CCK-8 assay measured HGF quantity on the surface after 24 h of culture (*p* < 0.05, Tukey Test), and (**B**) live/dead assays were conducted 24 h post-cell-seeding, with live cells exhibiting green fluorescence due to calcein AM staining, and dead cells showing red fluorescence from ethidium homodimer-1. ZL, monolayered zirconia with 3 mol% yttria (3Y-TZP), IPS e.max ZirCAD LT; ZM, monolayered zirconia with 4 mol% yttria (4Y-TZP), IPS e.max ZirCAD MT; ZT, incisal zone of multilayered zirconia with 5 mol% yttria (5Y-TZP), IPS e.max ZirCAD MT Multi; ZP, transition zone of multilayered zirconia with 3 and 5 mol% yttria (3Y/5Y-TZP), IPS e.max ZirCAD Prime. All data are presented as mean ± standard deviation values. * denotes a significant difference at *p* < 0.05.

The viability and proliferation of HGF on various materials were measured using the CCK-8 assay after 24 h cell seeding. The experimental results show that the cell viability decreased gradually with age in all the groups (Figure 4A). After first aging, the cell viability was highest in ZL and ZT, followed by ZP and ZM. After second aging, the cell viability was highest in ZL, followed by ZT, ZP, and ZM. These results indicate that hydrothermal aging reduced cell viability (Table 8).

### 3.7. Live/Dead Staining

To evaluate the viability of HGF after 24 h cell seeding, a live/dead assay was employed, wherein live cells (green) and dead cells (red) were stained, as shown in Figure 4B. Before aging, the number of dead cells was minimal across all the groups. However, the number of dead cells increased after the first aging period. The ZM group exhibited the lowest cell viability, followed by the ZL, ZT, and ZP groups. After the second aging period, a considerable increase in the number of dead cells was exhibited in the ZM group. These results demonstrate that prolonged hydrothermal aging reduces the biocompatibility of ZM more than the ZL, ZT, and ZP materials, thereby highlighting the considerable differences in their long-term suitability for biomedical applications.

### 3.8. Immunofluorescence Analysis of Cellular Cytoskeletal Organization

**Figure 5:**
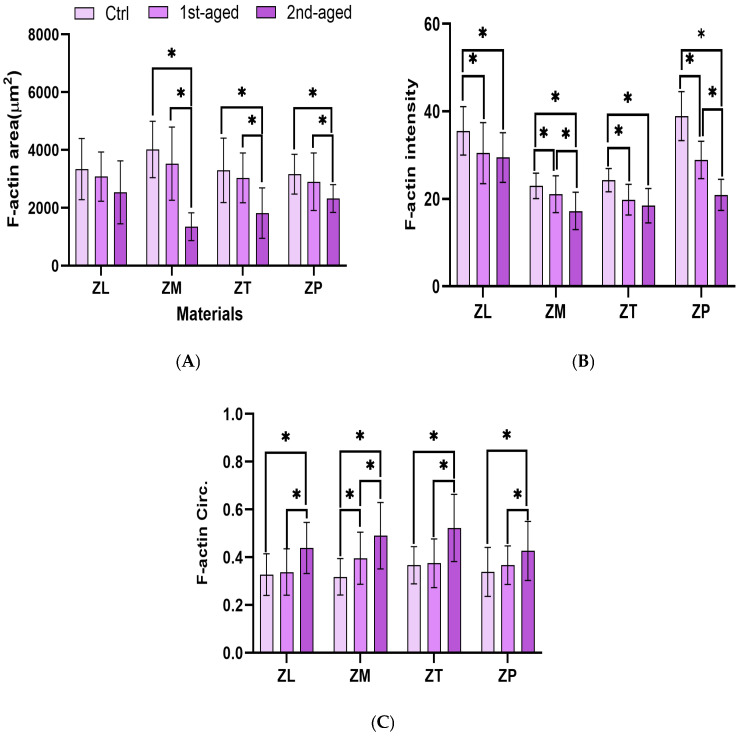
(**A**) F-actin area; (**B**) F-actin intensity; and (**C**) F-actin circ. (circularity). ZL, monolayered zirconia with 3 mol% yttria (3Y-TZP), IPS e.max ZirCAD LT; ZM, monolayered zirconia with 4 mol% yttria (4Y-TZP), IPS e.max ZirCAD MT; ZT, incisal zone of multilayered zirconia with 5 mol% yttria (5Y-TZP), IPS e.max ZirCAD MT Multi; ZP, transition zone of multilayered zirconia with 3 and 5 mol% yttria (3Y/5Y-TZP), IPS e.max ZirCAD Prime. All data are presented as mean ± standard deviation values. * denotes a significant difference at *p* < 0.05.

To analyze the morphology of the cells based on the material used, cell spreading and size were examined using fluorescence microscopy (Figure 5). The HGFs were stained using red phalloidin (F-actin) and green SYTOX (nuclear). In all the groups, the F-actin area decreased with age compared to the control group. The ZM group exhibited the largest F-actin area before aging and after the first aging cycle, but it showed the smallest area after the second aging cycle. The F-actin intensity also decreased with aging compared to that in the control group. Before aging, the ZP group had the highest intensity, while the ZM group had the lowest. After the first aging, the ZL group had the highest intensity, while the ZT group had the lowest. Following the second aging cycle, the ZL group exhibited the highest intensity, whereas the ZM group exhibited the lowest. Coupled with the decrease in the cell size and intensity with aging, a more rounded shape was observed in the cell morphology. These findings suggest that hydrothermal aging considerably impacts the cytoskeletal structure and adhesion properties of HGFs on different materials.

### 3.9. Immunofluorescence Analysis of Cell Focal Adhesion

**Figure 6:**
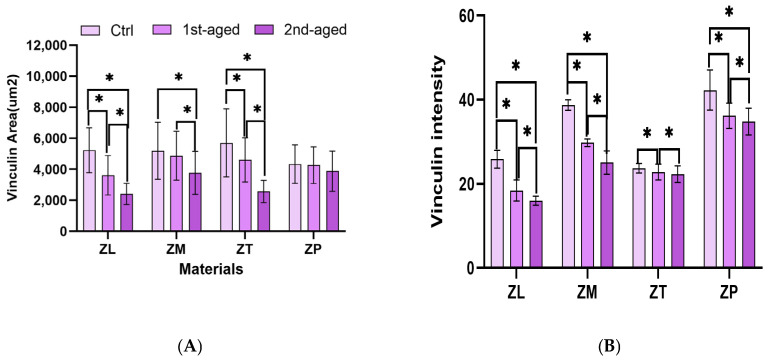
(**A**) Vinculin area; and (**B**) vinculin intensity. ZL, monolayered zirconia with 3 mol% yttria (3Y-TZP), IPS e.max ZirCAD LT; ZM, monolayered zirconia with 4 mol% yttria (4Y-TZP), IPS e.max ZirCAD MT; ZT, incisal zone of multilayered zirconia with 5 mol% yttria (5Y-TZP), IPS e.max ZirCAD MT Multi; ZP, transition zone of multilayered zirconia with 3 and 5 mol% yttria (3Y/5Y-TZP), IPS e.max ZirCAD Prime. All data are presented as mean ± standard deviation values. * denotes a significant difference at *p* < 0.05.

To investigate the adhesion properties of the HGF cells on the experimental surfaces, immunofluorescence staining was used to visualize the adhesion-related protein vinculin, which is a key component of the membrane skeleton (Figure 6). Starting from the highest value in the control group, both ZL and ZT considerably decreased to the lowest value with aging, while ZM considerably decreased only from the control to the second group. The ZP group showed no considerable decrease at any age. The vinculin area in the ZP group did not exhibit a considerable change during hydrothermal aging, thereby suggesting that the surface properties influencing cell adhesion might be relatively stable throughout the aging process for this particular yttria content. Conversely, the ZL and ZT groups considerably decreased in the vinculin area with aging. The considerable decrease from the control to the first-aged (*p* < 0.001) and form the first- to the second-aged groups (*p*† < 0.001) indicates that aging impacts the surface properties, thereby reducing cell adhesion over time. ZM exhibited similar patterns to those of ZP, while ZL exhibited those similar to ZT. All the specimens exhibited a considerable decrease in vinculin intensity from the control to the first-age group and from the control to the second-age group. ZT and ZP showed no considerable decrease from the first to the second age. ZP started with the highest vinculin intensity among all the groups at (42.29 ± 4.78). After the first aging, there was a significant decrease to 36.18 ± 3.00, and further aging reduced it to 34.81 ± 3.18, which remained the highest across all the specimens. The relatively high vinculin intensity in the ZP could be attributed to an optimal combination of the surface properties owing to the transition between 3% and 5% yttria content, thereby promoting better protein adsorption and cell adhesion. ZT exhibited the lowest vinculin intensity in the control group (23.71 ± 1.12). After the first aging phase, there was a slight, though statistically significant decrease to 22.82 ± 1.87 (*p* = 0.015). The second aging phase did not result in a statistically considerable change from the first aging phase (*p*† = 0.149), with a mean intensity of 22.29 ± 1.99. This suggests that the first aging process could have slightly affected the surface properties that contribute to cell adhesion. However, further aging did not considerably exacerbate this effect (Table 9 and Table 10).

## 4. Discussion

The effects of different yttrium oxide contents on the optical properties, surface properties, and biocompatibility of zirconia during hydrothermal aging are crucial for understanding the long-term performance and reliability of zirconia-based dental restoratives in the oral environment. Studies investigating the impact of aging effects on the optical properties and the microstructure of translucent zirconia offer crucial insights into how different yttria contents affect these key material characteristics during hydrothermal aging [49].

Different yttrium oxide contents considerably impact the color stability, which is indicative of its vulnerability to low-temperature degradation [50]. The observed color changes (ΔE_00_) across the different specimens are key indicators of the color stability of zirconia. Recent research indicates that the CIEDE2000 (ΔE_00_) color difference formula offers more precise correlations between perceived and calculated color differences than the older CIELAB (ΔE) method, enhancing its suitability for dental applications [51,52]. Studies have demonstrated that the human eye cannot discern subtle differences in color in dental materials when the numerical differences are minimal. Additionally, dentistry standards specify a CIEDE2000 perception threshold and acceptance threshold of ΔE_00_ = 0.8 and ΔE_00_ = 1.8, respectively [53]. A color match is considered excellent if ΔE_00_ is 0.8 or less and acceptable if it is greater than 0.8 but not exceeding 1.8 [54]. This research adopts ΔE_00_ = 1.8 as the measure for color difference evaluation. For ZL, ZM, ZT, and ZP, the color differences were clinically acceptable (ΔE_00_ = 1.8).

In this study, the TP was used to quantitatively assess how well zirconia materials mimic the optical properties of natural teeth, which is crucial for anterior restorations, where esthetics are critical. The TP values showed that transparency increased with a higher yttrium content, which corresponds with findings from previous studies [55]. ZT exhibited excellent transparency, thereby highlighting its suitability for anterior restorations. The impact of yttrium on the translucency and light transmission varied among the different types of zirconia, thereby reflecting their unique compositions and structural characteristics. This suggests that while all materials maintain some level of color stability, certain types of zirconia may be more suitable for anterior restorations. Hydrothermal aging caused minor alterations in the color parameters of zirconia dental materials, while the optical characteristics of translucency and color stability were largely preserved. However, slight variations among the different zirconia types emphasize the importance of selecting materials based on specific esthetic requirements.

These findings suggest that, depending on the clinical requirements, a material with balanced yttria content, such as ZP (3Y/5Y-TZP), may offer the best compromise between color stability and translucency. The performance of ZP across both of these parameters makes it particularly well-suited for achieving both esthetic and durable outcomes in dental restorations, while mitigating the adverse effects of aging [56]. Therefore, when selecting zirconia, clinicians should take into consideration the specific esthetic and functional needs of the patient, as well as the expected long-term exposure to varying conditions in the oral environment [57].

Exposure of zirconia to moist and warm environments accelerates its phase transformation [58]. Notably, the conversion from the tetragonal to monoclinic phase results in volumetric expansion, which likely explains the observed increase in the surface roughness [59]. Further, low-temperature degradation may weaken the grain boundary, thereby contributing to the increased surface roughness post-aging [60].

The considerable increase in the surface roughness from the control to the first-aged group across all the specimens indicates the initial susceptibility of zirconia to aging. However, the less pronounced and considerable increase from the first- to second-age group suggests a potential saturation point in the aging process, wherein additional aging reduces changes in the surface roughness. This could be due to the stabilization of the phase transformation over time, thereby requiring further studies. Notably, the considerable increase in the roughness parameters between the first- and second-age groups for ZT and ZP may indicate differing rates or extents of phase transformation or microcrack development within these particular groups. The variability in these rates may be attributed to the intrinsic properties of zirconia, such as the dopant levels, grain size, and presence of residual stresses [57], which require extensive analysis. Although the surface roughness increased in all the specimens after hydrothermal aging, all the values remained clinically acceptable (*R_a_* < 0.2 mm) [61]. These findings offer valuable insights into the aging behavior of zirconia, which is crucial for assessing the longevity and performance of zirconia-based components in clinical settings. Ongoing research is crucial to fully elucidate the aging mechanisms, thereby allowing for the optimization of the application of zirconia and development of strategies to mitigate the aging effects. At this point, there exist important clinical implications for wear resistance and bacterial adhesion. Increased surface roughness can elevate the risk of wear on opposing teeth and promote bacterial adhesion, potentially leading to complications, such as peri-implantitis or secondary caries. This highlights the need to consider both the esthetic and functional properties, including wear resistance and bacterial adhesion. To mitigate these effects, additional surface treatments like polishing are recommended to maintain smoothness.

The water contact angle (WCA) assessments revealed a decrease in the contact angles, thereby reflecting an increase in hydrophilicity. This enhanced wettability improves protein adsorption, which is crucial for cell adhesion, by optimizing the surface wetting and distribution of adhesion molecules [62]. This consistent effect of aging on the water contact angle suggests that the aging process modifies the surface of the material, thereby influencing its interaction with water molecules [63]. These findings suggest that the aging process alters the microtopography of the zirconia surface, thereby influencing its mechanical properties and performance in clinical applications. Increased hydrophilicity following aging has a complex effect on the clinical relevance of zirconia. Although enhanced hydrophilicity is generally associated with improved initial wettability and protein interaction, this study found a decrease in cell adhesion despite increased hydrophilicity. This suggests that excessively high hydrophilicity may hinder stable cell attachment, thereby potentially degrading long-term biocompatibility and integration with gingival tissues. Balancing optimal wettability with the surface microstructure is essential for achieving successful tissue integration in the long term.

The CCK-8 and live/dead assays revealed that cell viability decreased with age. This suggests that even minor changes in the zirconia surface, such as an increase in the specific surface roughness, may hamper cell adhesion. However, the majority of human gingival fibroblasts (HGF) remained attached to the surface of the material, thereby indicating that zirconia can maintain a considerable level of biocompatibility even after aging. Although an increase in surface roughness can adversely impact cell adhesion, it can also create a microstructure that supports initial cell adhesion [64,65]. Hence, the impact of zirconia aging on cell adhesion is complex, thereby reflecting a balance between surface property changes and cellular responses.

Distinct patterns of the F-actin characteristics across different age groups and conditions highlight the impact of aging on cell morphology and function [66]. ZM exhibited the largest F-actin area values in the control and first-age groups but exhibited the smallest in the second-age group, thereby indicating a decrease in cell spreading with prolonged aging. No considerable changes in the area from the control to the first age across all the groups suggest maintained cell-spreading capacity up to a specific age threshold, after which a decline becomes evident.

The analysis revealed that ZM had the lowest intensity values for the control and second-aged groups, thereby indicating a decrease in the F-actin expression with age, including that in the structural integrity and mechanical stability of the cells [63]. Circularity data showed that ZM had the lowest value in the control group, thereby indicating a less rounded morphology, contrary to the highest value in the first-aged group, which suggests that cells become more rounded with aging.

These findings suggest that cell spreading decreases with age, with cells adopting a more rounded morphology, thereby indicating a decrease in cell function and plasticity. Changes in vinculin expression, notably in the ZP, indicate a shift in the cell attachment dynamics post-aging, thereby enhancing robust long-term clinical integration.

This study highlights the importance of a balanced approach in dental material engineering to enhance initial cell adhesion while ensuring long-term viability and structural integrity. The transitional yttria content in the ZP maintains favorable surface properties for cell adhesion, even as the material ages, thereby balancing phase stability and surface roughness.

The role of surface topography and roughness significantly influences cell proliferation and adhesion in zirconia. The surface roughness increased across all the zirconia types after hydrothermal aging, with the highest values observed in ZL (3Y-TZP). Increased surface roughness may enhance initial cell attachment by providing greater surface area, but excessive roughness can negatively affect long-term cell proliferation and stable adhesion. The differences in the F-actin area and vinculin expression further illustrate the impact of surface changes on cellular behavior. ZM initially showed favorable F-actin expression, but a reduction in F-actin and vinculin levels after aging suggested diminished cytoskeletal integrity and cell attachment stability. In contrast, ZP (3Y/5Y-TZP) maintained more stable vinculin levels, indicating better compatibility with cell adhesion. These findings underscore the importance of optimizing the surface roughness and microstructure to enhance both early and long-term adhesion, ensuring successful integration with gingival tissues.

Both 3Y/5Y-TZP and 3Y-TZP demonstrated resistance to strength degradation while maintaining their color stability and surface integrity after aging. Gradient materials with different yttria contents (3Y/5Y-TZP) showed no disadvantages in terms of mechanical properties or biocompatibility compared with single-component materials, thereby suggesting an optimized balance between transparency, biocompatibility, and mechanical strength without compromising aging resistance. Future studies are required to further explore the relationship between the aging microstructural changes on zirconia surfaces and cell adhesion mechanisms. Long-term clinical studies must be conducted to determine whether these experimental results are reproducible in an actual oral environment.

## 5. Conclusions

This study acknowledges several limitations that should be considered for a balanced interpretation and to guide future research. First, the hydrothermal aging process, conducted at 134 °C and 0.2 MPa for set durations of 0, 5, and 10 h, may not fully replicate the broad range of temperature variations and mechanical stresses experienced over extended periods in the oral cavity. Additionally, while in vitro biocompatibility assessments using HGFs offer valuable insights, they do not capture the complexity of in vivo conditions, where multiple cell types, immune responses, and interactions with saliva and microbial flora play a significant role. Lastly, although this study focused on phase transitions between tetragonal and monoclinic structures during aging, it did not explore how these transitions might affect the mechanical properties of zirconia under cyclic mechanical loading, such as those experienced during mastication.

### 5.1. Optical Properties

3Y/5Y-TZP demonstrated the highest color stability and exhibited superior translucency with lower contrast ratio values. In comparison, 5Y-TZP showed lower color stability, though it remained within clinically acceptable limits.

### 5.2. Surface Properties

The surface roughness of 3Y-TZP increased following aging, but the values remained within clinically acceptable limits.

### 5.3. Cellular Properties

4Y-TZP showed the lowest cell viability and exhibited the poorest performance in the F-actin analysis. Live HGF cells were observed throughout all the aging periods, and 3Y/5Y-TZP demonstrated the highest vinculin values and cell adhesion across all the aging stages.

## Figures and Tables

**Table 1:** Material composition and manufacturer details.

Product Name	Shade (Lot No.)	Composition
IPS e.max ZirCAD LT	A2 (Z02R0D)	3Y-TZP Zirconium oxide (88.0–95.5 wt%) Yttrium oxide (4.5–6.0 wt%) Hafnium oxide (≤5.0 wt%) Aluminum oxide (≤1.0 wt%) Other oxides (≤1.0 wt%)
IPS e.max ZirCAD MT	A2 (Z01WN7)	4Y-TZP Zirconium oxide (86.0–93.5 wt%) Yttrium oxide (6.5–8.0 wt%) Hafnium oxide (≤5.0 wt%) Aluminum oxide (≤1.0 wt%) Other oxides (≤1.0 wt%)
IPS e.max ZirCAD MT Multi	A2 (Z04PZS)	4Y-TZP/5Y-TZP Zirconium oxide (86.0–93.5 wt%) Yttrium oxide (6.5–8.0 wt%) Hafnium oxide (≤5.0 wt%) Aluminum oxide (≤1.0 wt%) Other oxides (≤1.0 wt%)
IPS e.max ZirCAD Prime	A2 (Z05C6L)	3Y-TZP/5Y-TZP Zirconium oxide (88.0–95.5 wt%) Yttrium oxide (4.5–7.0 wt%) Hafnium oxide (≤5.0 wt%) Aluminum oxide (≤1.0 wt%) Other oxides (≤1.5 wt%)

Manufacturer’s published values. Y-TZP: yttria stabilized-tetragonal zirconia polycrystals.

**Table 2:** Abbreviations for the experimental material groups.

Zone of Translucent Zirconia	Type	MaterialCode	Groups		
			Control	First Aged	Second Aged
3Y-TZP of IPS e.max ZirCAD LT	Monolayerd	ZL	ZLC	ZLAF	ZLAS
4Y-TZP of IPS e.max ZirCAD MT	Monolayerd	ZM	ZMC	ZMAF	ZMAS
5Y-TZP of IPS e.max ZirCAD MT Multi	Multilayered (Incisal zone)	ZT	ZTC	ZTAF	ZTAS
3Y/5Y-TZP of IPS e.max ZirCAD Prime	Multilayered (Transition zone)	ZP	ZPC	ZPAF	ZPAS

ZL, 3Y-TZP of IPS e.max ZirCAD LT; ZM, 4Y-TZP of IPS e.max ZirCAD MT; ZT, 5Y-TZP of IPS e.max ZirCAD MT Multi; ZP, 3Y/5Y-TZP of IPS e.max ZirCAD Prime.

**Table 3:** Mean ± SD values and statistical analysis of CIELab (black background, SCI).

Value	Group	Mean ± SD					
		Control	First Aged	*p*	Second Aged	*p* †	*p* ‡
L*	ZL	92.79 ± 0.24 ^a^	92.68 ± 0.32 ^a^	0.131	92.58 ± 0.34 ^a^	0.009 *	0.236
	ZM	85.82 ± 0.50 ^b^	85.81 ± 0.49 ^b^	0.948	85.22 ± 1.06 ^b^	0.008 *	0.008 *
	ZT	81.22 ± 3.02 ^c^	81.12 ± 2.51 ^c^	0.887	80.97 ± 2.59 ^c^	0.730	0.822
	ZP	93.43 ± 0.36 ^a^	93.22 ± 0.51 ^a^	0.075	92.65 ± 2.50 ^a^	0.100	0.226
a*	ZL	−1.17 ± 0.18 ^a^	−1.18 ± 0.20 ^a^	0.819	−1.24 ± 0.25 ^a^	0.250	0.361
	ZM	−1.25 ± 0.11 ^a^	−1.29 ± 0.15 ^b^	0.196	−1.23 ± 0.15 ^a^	0.626	0.118
	ZT	−1.86 ± 0.11 ^b^	−1.84 ± 0.08 ^c^	0.550	−1.85 ± 0.08 ^b^	0.839	0.642
	ZP	0.21 ± 0.05 ^c^	0.20 ± 0.06 ^d^	0.418	0.05 ± 0.48 ^c^	0.081	0.105
b*	ZL	7.56 ± 0.23 ^a^	7.76 ± 0.20 ^a^	<0.001 *	7.79 ± 0.28 ^a^	<0.001 *	0.592
	ZM	10.53 ± 0.39 ^b^	10.50 ± 0.43 ^b^	0.734	10.31 ± 0.48 ^b^	0.050 *	0.111
	ZT	5.15 ± 0.81 ^c^	5.27 ± 0.80 ^c^	0.575	4.91 ± 0.80 ^c^	0.234	0.081
	ZP	8.36 ± 0.41 ^d^	8.36 ± 0.46 ^d^	0.946	8.67 ± 0.59 ^d^	0.024 *	0.027 *
C*	ZL	7.65 ± 0.20 ^a^	7.85 ± 0.18 ^a^	<0.001 *	7.90 ± 0.26 ^a^	<0.001 *	0.435
	ZM	10.61 ± 0.38 ^b^	10.58 ± 0.41 ^b^	0.765	10.38 ± 0.47 ^b^	0.044 *	0.090
	ZT	5.49 ± 0.71 ^c^	5.59 ± 0.73 ^c^	0.579	5.25 ± 0.72 ^c^	0.205	0.073
	ZP	8.37 ± 0.41 ^d^	8.36 ± 0.46 ^d^	0.938	8.68 ± 0.61 ^d^	0.023 *	0.025 *
h	ZL	98.86 ± 1.54 ^a^	98.70 ± 1.60 ^a^	0.694	99.07 ± 2.02 ^a^	0.655	0.436
	ZM	96.77 ± 0.80 ^b^	97.050 ± 1.03 ^b^	0.244	96.84 ± 1.09 ^b^	0.780	0.444
	ZT	110.35 ± 4.30 b	109.70 ± 3.66 c	0.532	111.17 ± 3.87 c	0.440	0.137
	ZP	88.56 ± 0.39 c	88.64 ± 0.43 d	0.466	89.52 ± 2.81 d	0.073	0.099

ZL, 3Y-TZP of IPS e.max ZirCAD LT; ZM, 4Y-TZP of IPS e.max ZirCAD MT; ZT, 5Y-TZP of IPS e.max ZirCAD MT Multi; ZP, 3Y/5Y-TZP of IPS e.max ZirCAD Prime. Significant differences between the values in each column are denoted by different superscript letters, which indicate the statistical significance (*p* < 0.05). *p* values were calculated using the independent *t*-test results of the samples in the control and first-age groups. *p* † values were calculated using the independent *t*-test results of the samples in the control and secondary age groups. *p* ‡ values were calculated using the independent *t*-test results of the samples in the first- and second-age groups. * denotes a significant difference at *p* < 0.05.

**Table 4:** Mean ± SD values and statistical analysis of ΔE and ΔE_00_ (black background, SCI).

Value	Materials	Control–First Aged	Control–Second Aged	*p*	First aged–Second Aged	*p* †	*p* ‡
ΔE	ZL	0.09 ± 0.06 ^a^	0.22 ± 0.14 ^a^	<0.001 *	0.16 ± 0.11 ^ac^	0.006 *	0.103
	ZM	0.35 ± 0.26 ^b^	0.38 ± 0.21 ^ab^	0.706	0.33 ± 0.27 ^bd^	0.705	0.433
	ZT	0.25 ± 0.18 ^bc^	0.53 ± 0.49 ^b^	0.006 *	0.42 ± 0.20 ^b^	0.001 *	0.228
	ZP	0.16 ± 0.15 ^ac^	0.31 ± 0.21 ^a^	0.002 *	0.25 ± 0.17 ^cd^	0.030 *	0.226
ΔE_00_	ZL	0.30 ± 0.09 ^ad^	0.39 ± 0.18 ^a^	0.02 *	0.39 ± 0.16 ^a^	0.010 *	0.960
	ZM	0.43 ± 0.22 ^b^	0.49 ± 0.18 ^ac^	0.201	0.41 ± 0.21 ^a^	0.726	0.088
	ZT	0.36 ± 0.17 ^ab^	0.61 ± 0.45 ^bc^	0.008 *	0.44 ± 0.11 ^a^	0.030 *	0.059
	ZP	0.25 ± 0.13 ^cd^	0.39 ± 0.15 ^a^	<0.001 *	0.35 ± 0.11 ^a^	0.003 *	0.271

ZL, 3Y-TZP of IPS e.max ZirCAD LT; ZM, 4Y-TZP of IPS e.max ZirCAD MT; ZT, 5Y-TZP of IPS e.max ZirCAD MT Multi; ZP, 3Y/5Y-TZP of IPS e.max ZirCAD Prime. Significant differences between the values in each column are denoted by different superscript letters, which indicate the statistical significance (*p* < 0.05). *p* values were calculated using the independent *t*-test results of the samples in the control and first-age groups. *p* † values were calculated using the independent *t*-test results of the samples in the control and secondary age groups. *p* ‡ values were calculated using the independent *t*-test results of the samples in the first- and second-age groups. * denotes a significant difference at *p* < 0.05.

**Table 5:** Mean ± SD values and statistical analysis of optical properties.

Value	Group	Mean ± SD					
		Control	First Aged	*p*	Second Aged	*p* †	*p* ‡
At	ZL	38.79 ± 4.97 ^a^	38.13 ± 1.17 ^a^	0.482	37.10 ± 1.05 ^a^	0.074	<0.001 *
	ZM	47.38 ± 0.63 ^b^	47.36 ± 0.80 ^b^	0.939	46.52 ± 1.39 ^b^	0.003 *	0.006 *
	ZT	57.33 ± 3.78 ^c^	57.05 ± 3.09 ^c^	0.750	57.03 ± 3.30 ^c^	0.745	0.986
	ZP	39.10 ± 0.39 ^a^	39.06 ± 0.69 ^a^	0.765	38.75 ± 0.49 ^d^	0.004 *	0.054
TP	ZL	0.11 ± 0.02 ^a^	0.11 ± 0.02 ^a^	0.271	0.10 ± 0.01 ^a^	0.013 *	0.220
	ZM	2.32 ± 0.46 ^b^	2.15 ± 0.25 ^b^	0.090	2.01 ± 0.34 ^b^	0.005 *	0.084
	ZT	5.05 ± 0.83 ^c^	4.96 ± 1.04 ^c^	0.713	4.82 ± 1.03 ^c^	0.355	0.613
	ZP	0.13 ± 0.01 ^a^	0.12 ± 0.01 ^a^	0.005 *	0.12 ± 0.03 ^a^	0.035 *	0.453
CR	ZL	1.00 ± 0.01 ^a^	1.00 ± 0.00 ^a^	0.101	1.00 ± 0.00 ^a^	0.111	0.220
	ZM	0.96 ± 0.01 ^b^	0.97 ± 0.01 ^b^	0.004 *	0.97 ± 0.00 ^b^	0.109	0.044 *
	ZT	0.89 ± 0.02 ^c^	0.90 ± 0.03 ^c^	0.690	0.91 ± 0.04 ^c^	0.177	0.326
	ZP	1.00 ± 0.00 ^a^	1.00 ± 0.00 ^a^	0.126	1.00 ± 0.11 ^a^	0.052	0.058

ZL, 3Y-TZP of IPS e.max ZirCAD LT; ZM, 4Y-TZP of IPS e.max ZirCAD MT; ZT, 5Y-TZP of IPS e.max ZirCAD MT Multi; ZP, 3Y/5Y-TZP of IPS e.max ZirCAD Prime. Significant differences between the values in each column are denoted by different superscript letters, which indicate the statistical significance (*p* < 0.05). *p* values were calculated using the independent *t*-test results of the samples in the control and first-age groups. *p* † values were calculated using the independent *t*-test results of the samples in the control and secondary age groups. *p* ‡ values were calculated using the independent *t*-test results of the samples in the first- and second-age groups. * denotes a significant difference at *p* < 0.05.

**Table 6:** Mean ± SD values and statistical analysis of the surface roughness (*R_a_, R_q_, S_a_*, and *S_q_*).

Value	Group	Mean ± SD (nm)					
		Control	First Aged	*p*	Second Aged	*p* †	*p* ‡
*R_a_*	ZL	3.95 ± 0.34 ^a^	6.70 ± 0.62 ^a^	<0.001 *	6.95 ± 0.27 ^a^	<0.001 *	0.260
	ZM	3.07 ± 0.43 ^b^	3.66 ± 0.94 ^b^	0.005 *	3.99 ± 0.71 ^b^	<0.001 *	0.154
	ZT	2.95 ± 0.86 ^b^	3.38 ± 0.42 ^b^	0.026 *	3.87 ± 0.37 ^b^	<0.001 *	<0.001 *
	ZP	3.31 ± 0.66 ^b^	5.33 ± 0.55 ^c^	<0.001 *	5.87 ± 0.59 ^c^	<0.001 *	<0.001 *
*R_q_*	ZL	5.44 ± 0.85 ^a^	8.82 ± 1.08 ^a^	<0.001 *	8.90 ± 1.04 ^a^	<0.001 *	0.781
	ZM	4.05 ± 0.53 ^b^	5.08 ± 1.12 ^b^	<0.001 *	5.40 ± 1.06 ^b^	<0.001 *	0.299
	ZT	3.90 ± 1.12 ^b^	4.51 ± 0.55 ^b^	0.016 *	5.34 ± 0.64 ^b^	<0.001 *	<0.001 *
	ZP	4.36 ± 0.83 ^b^	6.84 ± 0.73 ^c^	<0.001 *	7.58 ± 0.67 ^c^	<0.001 *	<0.001 *
*S_a_*	ZL	4.41 ± 1.25 ^a^	7.3 ±0.76 ^a^	<0.001 *	7.62 ± 2.03 ^a^	<0.001 *	0.462
	ZM	3.20 ± 0.38 ^b^	3.92 ± 0.87 ^b^	<0.001 *	4.10 ± 0.72 ^b^	<0.001 *	0.418
	ZT	2.98 ± 0.86 ^b^	3.40 ± 0.41 ^b^	0.026 *	3.91 ± 0.41 ^b^	<0.001 *	<0.001 *
	ZP	3.33 ± 0.68 ^b^	5.34 ± 0.56 ^c^	<0.001 *	5.90 ± 0.58 ^c^	<0.001 *	<0.001 *
*S_q_*	ZL	5.86 ± 1.62 ^a^	9.30 ± 1.03 ^a^	<0.001 *	9.64 ± 2.34 ^a^	<0.001 *	0.488
	ZM	4.27 ± 0.49 ^b^	5.34 ± 1.07 ^b^	<0.001 *	5.60 ± 1.07 ^b^	<0.001 *	0.384
	ZT	3.91 ± 1.10 ^b^	4.51 ± 0.55 ^b^	0.016 *	5.36 ± 0.65 ^b^	<0.001 *	<0.001 *
	ZP	4.37 ± 0.83 ^b^	6.86 ± 0.73 ^c^	<0.001 *	7.60 ± 0.64 ^c^	<0.001 *	<0.001 *

ZL, 3Y-TZP of IPS e.max ZirCAD LT; ZM, 4Y-TZP of IPS e.max ZirCAD MT; ZT, 5Y-TZP of IPS e.max ZirCAD MT Multi; ZP, 3Y/5Y-TZP of IPS e.max ZirCAD Prime. Significant differences between the values in each column are denoted by different superscript letters, which indicate the statistical significance (*p* < 0.05). *p* values were calculated using the independent *t*-test results of the samples in the control and first-age groups. *p* † values were calculated using the independent *t*-test results of the samples in the control and secondary age groups. *p* ‡ values were calculated using the independent *t*-test results of the samples in the first- and second-age groups. * denotes a significant difference at *p* < 0.05.

**Table 7:** Mean ± SD values and statistical analysis of hydrophilicity.

Group	Mean ± SD (°)					
	Control	First Aged	*p*	Second Aged	*p* †	*p* ‡
ZL	50.18 ± 7.10 ^a^	42.22 ± 0.84 ^a^	<0.001 *	32.96 ± 2.06 ^a^	<0.001 *	<0.001 *
ZM	59.54 ± 4.75 ^b^	53.28 ± 3.15 ^b^	<0.001 *	52.73 ± 6.52 ^b^	<0.001 *	0.696
ZT	51.05 ± 6.72 ^ac^	50.42 ± 7.10 ^b^	0.757	50.36 ± 6.63 ^b^	0.707	0.975
ZP	54.57 ± 3.77 ^c^	51.05 ± 6.72 ^b^	0.022 *	50.42 ± 7.10 ^b^	0.018 *	0.757

ZL, 3Y-TZP of IPS e.max ZirCAD LT; ZM, 4Y-TZP of IPS e.max ZirCAD MT; ZT, 5Y-TZP of IPS e.max ZirCAD MT Multi; ZP, 3Y/5Y-TZP of IPS e.max ZirCAD Prime. Significant differences between the values in each column are denoted by different superscript letters, which indicate the statistical significance (*p* < 0.05). *p* values were calculated using the independent *t*-test results of the samples in the control and first-age groups. *p* † values were calculated using the independent *t*-test results of the samples in the control and secondary age. groups. *p* ‡ values were calculated using the independent *t*-test results of the samples in the first- and second-age groups. * denotes a significant difference at *p* < 0.05.

**Table 8:** Mean ± SD values and statistical analysis of optical density (CCK).

Group	Mean ± SD (AU)					
	Control	First Aged	*p*	Second Aged	*p* †	*p* ‡
ZL	0.60 ± 0.01 ^a^	0.60 ± 0.01 ^a^	0.744	0.58 ± 0.01 ^a^	<0.001 *	<0.001 *
ZM	0.57 ± 0.01 ^b^	0.37 ± 0.01 ^b^	<0.001 *	0.19 ± 0.00 ^b^	<0.001 *	<0.001 *
ZT	0.67 ± 0.01 ^c^	0.60 ± 0.01 ^a^	<0.001 *	0.46 ± 0.01 ^c^	<0.001 *	<0.001 *
ZP	0.69 ± 0.01 ^d^	0.56 ± 0.01 ^c^	<0.001 *	0.38 ± 0.01 ^d^	<0.001 *	<0.001 *

ZL, 3Y-TZP of IPS e.max ZirCAD LT; ZM, 4Y-TZP of IPS e.max ZirCAD MT; ZT, 5Y-TZP of IPS e.max ZirCAD MT Multi; ZP, 3Y/5Y-TZP of IPS e.max ZirCAD Prime. Significant differences between the values in each column are denoted by different superscript letters, which indicate the statistical significance (*p* < 0.05). *p* values were calculated using the independent *t*-test results of the samples in the control and first-age groups. *p* † values were calculated using the independent *t*-test results of the samples in the control and secondary age groups. *p* ‡ values were calculated using the independent *t*-test results of the samples in the first- and second-age groups. * denotes a significant difference at *p* < 0.05.

**Table 9:** Mean ± SD values and statistical analysis focal adhesion (vinculin area).

Group	Mean ± SD (μm)					
	Control	First Aged	*p*	Second Aged	*p* †	*p* ‡
ZL	5227.33 ± 1445.83 ^ab^	3615.57 ± 1268.43 ^a^	<0.001 *	2410.16 ± 685.75 ^a^	<0.001 *	<0.001 *
ZM	5193.43 ± 1839.31 ^ab^	4877.70 ± 1575.66 ^b^	0.478	3769.71 ± 1383.40 ^b^	0.001 *	0.005 *
ZT	5699.64 ± 2194.54 ^a^	4603.39 ± 1428.83 ^b^	0.026 *	2567.49 ± 712.52 ^a^	<0.001 *	<0.001 *
ZP	4332.49 ± 1241.59 ^b^	4264.01 ± 1176.29 ^ab^	0.827	3883.62 ± 1295.79 ^b^	0.176	0.239

ZL, 3Y-TZP of IPS e.max ZirCAD LT; ZM, 4Y-TZP of IPS e.max ZirCAD MT; ZT, 5Y-TZP of IPS e.max ZirCAD MT Multi; ZP, 3Y/5Y-TZP of IPS e.max ZirCAD Prime. Significant differences between the values in each column are denoted by different superscript letters, which indicate the statistical significance (*p* < 0.05). *p* values were calculated using the independent t-test results of the samples in the control and first-age groups. *p* † values were calculated using the independent t-test results of the samples in the control and secondary age groups. *p* ‡ values were calculated using the independent t-test results of the samples in the first- and second-age groups. * denotes a significant difference at *p* < 0.05.

**Table 10:** Mean ± SD values and statistical analysis focal adhesion (vinculin intensity).

Group	Mean ± SD (AU)					
	Control	First Aged	*p*	Second Aged	*p* †	*p* ‡
ZL	25.86 ± 2.14 ^a^	18.44 ± 2.49 ^a^	<0.001 *	16.01 ± 1.09 ^a^	<0.001 *	<0.001 *
ZM	38.75 ± 1.25 ^b^	29.77 ± 0.91 ^b^	<0.001 *	25.05 ± 2.78 ^b^	<0.001 *	<0.001 *
ZT	23.71 ± 1.12 ^c^	22.82 ± 1.87 ^c^	0.029 *	22.29 ± 1.99 ^c^	0.001 *	0.298
ZP	42.29 ± 4.78 ^d^	36.18 ± 3.00 ^d^	<0.001 *	34.81 ± 3.18 ^d^	<0.001 *	0.092

ZL, 3Y-TZP of IPS e.max ZirCAD LT; ZM, 4Y-TZP of IPS e.max ZirCAD MT; ZT, 5Y-TZP of IPS e.max ZirCAD MT Multi; ZP, 3Y/5Y-TZP of IPS e.max ZirCAD Prime. Significant differences between the values in each column are denoted by different superscript letters, which indicate the statistical significance (*p* < 0.05). *p* values were calculated using the independent *t*-test results of the samples in the control and first-age groups. *p* † values were calculated using the independent *t*-test results of the samples in the control and secondary age groups. *p* ‡ values were calculated using the independent *t*-test results of the samples in the first- and second-age groups. * denotes a significant difference at *p* < 0.05.

## Data Availability

The original contributions presented in the study are included in the article, further inquiries can be directed to the corresponding authors.
